# Wild type human tau is more prone to phosphorylation than P301L‐tau in the presence of amyloidosis in mouse brain

**DOI:** 10.1002/alz70855_107183

**Published:** 2025-12-24

**Authors:** Dave Morgan, Dylan Finneran, Briana G Jackman, Taylor Desjarlais, Marcia N. Gordon

**Affiliations:** ^1^ Michigan State University, Grand Rapids, MI, USA

## Abstract

**Background:**

We have previously reported that APP+PS1 mice can enhance tauopathy when injected with a virus over‐expressing P301L tau. Here we compare the extent of tauopathy produced by the aggregation prone human P301L tau with wild type tau in the presence of mature amyloidosis.

**Method:**

Ten‐month‐old transgenic mice over‐expressing mutant amyloid precursor protein and presenilin 1 (APP+PS1) were injected intravenously with AAV.Cap‐B10 over‐expressing either wild‐type (AAV‐WT) or P301L (AAV‐P301L) 4R2N human tau. Half of the mice were behaviorally assessed four months post‐injection and euthanized five months post‐injection. The remaining mice were euthanized nine months post‐injection. Pathology was assessed by ELISA and immunohistochemistry.

**Result:**

By ELISA, measures of soluble total tau showed reduced levels in mice injected with AAV‐WT compared with AAV‐P301L at both five‐ and nine‐month survival times. However, in both cohorts, there was significantly more soluble pS396 tau in AAV‐WT mice relative to AAV‐P301L, while soluble pS199 tau was significantly increased in AAV‐WT mice in only the younger cohort. Measures of insoluble total tau were reduced in AAV‐WT compared to AAV‐P301L mice, yet insoluble phospho‐tau levels were similar in both cohorts. Similarly, histological measures of total tau (HT7) show reduced levels in mice injected with AAV‐WT relative to AAV‐P301L in both the cortex and hippocampus. However, we observed increased area staining of AT8‐positive phospho‐tau in mice injected with AAV‐WT compared with AAV‐P301L. In agreement with our protein measures, we observed significantly less human tau mRNA in mice injected with AAV‐WT compared to AAV‐P301L. ELISA measures of amyloid pathology were not significantly different between groups. Both AAV‐WT and AAV‐P301L mice displayed similar learning impairments compared to nontransgenic mice.

**Conclusion:**

Over‐expression of both wild‐type and P301L tau induced learning and memory deficits in middle‐aged APP+PS1 mice with mature amyloid pathology. While we observed less total tau in mice injected with AAV‐WT than AAV‐P301L, a greater fraction of the total available pool of tau was phosphorylated in mice over‐expressing wild‐type tau. These data suggest that although mutant P301L tau may be more prone to aggregation *in vitro*, WT‐tau is more prone to phosphorylation *in vivo* in the presence of amyloidosis.

